# Physiological Response of *Escherichia coli* O157:H7 Sakai to Dynamic Changes in Temperature and Water Activity as Experienced during Carcass Chilling*[Fn FN2]

**DOI:** 10.1074/mcp.M116.063065

**Published:** 2016-09-11

**Authors:** Thea King, Chawalit Kocharunchitt, Kari Gobius, John P. Bowman, Tom Ross

**Affiliations:** From the ‡CSIRO Agriculture and Food, North Ryde, NSW 2113, Australia;; §Food Safety Centre, Tasmanian Institute of Agriculture, School of Agricultural Science, University of Tasmania, Private Bag 54, Hobart TAS 7001, Australia;; ¶CSIRO Agriculture and Food, Werribee, VIC 3030, Australia

## Abstract

Enterohemeorrhagic *Escherichia coli* is a leading cause of foodborne illness, with the majority of cases linked to foods of bovine origin. Currently, no completely effective method for controlling this pathogen during carcass processing exists. Understanding how this pathogen behaves under those stress conditions experienced on the carcass during chilling in cold air could offer opportunities for development or improvement of effective decontamination processes. Therefore, we studied the growth kinetics and physiological response of exponential phase *E. coli* O157:H7 Sakai cultures upon an abrupt downshift in temperature and water activity (from 35 °C a_w_ 0.993 to 14 °C a_w_ 0.967). A parallel Biolog study was conducted to follow the phenotypic responses to 190 carbon sources. Exposure of *E. coli* to combined cold and water activity stresses resulted in a complex pattern of population changes. This pattern could be divided into two main phases, including adaptation and regrowth phases, based on growth kinetics and clustering analyses. The transcriptomic and proteomic studies revealed that *E. coli* exhibited a “window” of cell susceptibility (*i.e.* weaknesses) during adaptation phase. This included apparent DNA damage, the downregulation of molecular chaperones and proteins associated with responses to oxidative damage. However, *E. coli* also displayed a transient induction in the RpoE-controlled envelope stress response and activation of the master stress regulator RpoS and the Rcs phosphorelay system involved in colanic acid biosynthesis. Increased expression was observed for several genes and/or proteins involved in DNA repair, protein and peptide degradation, amino acid biosynthesis, and carbohydrate catabolism and energy generation. Furthermore, the Biolog study revealed reduced carbon source utilization during adaptation phase, indicating the disruption of energy-generating processes. This study provides insight into the physiological response of *E. coli* during exposure to combined cold and water activity stress, which could be exploited to enhance the microbiological safety of carcasses and related foods.

Escherichia coli O157 strains are prominent among pathogenic *E. coli* strains, referred to with various designations including enterohemorrhagic, verocytotoxin-producing, or Shiga-toxin-producing organisms and have become a significant cause of food-borne illness worldwide (for review see (1)). This pathogen causes human illness that ranges in severity from mild diarrhea to hemorrhagic colitis and the potentially life-threatening Hemolytic Uremic Syndrome (HUS). Although many food and dairy products have acted as vectors, the majority of reported cases appear to be attributed to the consumption of foods of bovine origin; with ground beef the most frequently identified vehicle (2). Cattle comprise a main reservoir of O157 and this organism can be found in their feces, rumen, hide, and derived carcasses (3–5). Foodborne disease and product recalls because of this organism continue to occur even though control measures have been under investigation for over 20 years (6). Because of the global nature of the red meat food supply chain, safety concerns with beef will continue and the challenges facing the beef industry will increase at the production and processing levels (7). Although intervention strategies are possible at many points along the production chain (6), an understanding of how *E. coli* O157 strains survive and proliferate on meat carcasses would permit the development of effective strategies to control growth or inactivate this pathogen.

On the meat carcass surface, several factors strongly influence the potential for, and rate of, growth of bacteria (8). In Australia, carcasses are traditionally chilled by circulation of cold air, which introduces another constraint aside from chill temperature stress, *i.e.* reduced water activity, because of surface drying of the carcass (8–10). These stressful environmental conditions have a significant impact on the growth and survival of microorganisms, including *E. coli* (11–14). Bacon *et al.* (15) reported that conventional chilling resulted in a reduction in *E. coli* of up to 2 log units over 24 to 36 h. Similarly, but of a smaller magnitude, air chilling was reported to cause a 0.3 to 0.7 log unit reduction in microbial populations on carcasses (16). Greig *et al.* (17) also analyzed the available literature on carcass chilling, and concluded that chilling alone can cause inactivation of *E. coli*. However, it has been demonstrated that such reductions following the chilling conditions were only transient, and were because of cell injury rather than cell death (14). This provides a “window” of opportunities in which novel “intervention” strategies could be applied to completely eliminate *E. coli* on carcasses during chilling. Therefore, a detailed understanding of the physiological responses of *E. col* to the temperature and water activity changes as occurs during air chilling is important in order to optimize the chilling process as an effective decontamination process during carcass processing.

Previously we undertook a baseline study employing both cDNA microarray and 2D-LC/MS/MS analyses to elucidate the transcriptional and proteomic responses of exponential phase *E. coli* O157:H7 strain Sakai grown under steady-state conditions, relevant to low temperature and water activity conditions experienced during carcass chilling (18). This study was extended to investigate the growth kinetics of this pathogen subjected to an abrupt downshift in water activity (a_w_ 0.993 to either a_w_ 0.980, 0.975, 0.970, 0.967, or 0.960) (19) and temperature (35 °C to either 20 °C, 17 °C, 14 °C or 10 °C) (20), as well as to examine the time-dependent alterations in its transcriptome and proteome during adaptation upon an abrupt downshift in a_w_ (from 0.993 to a_w_ 0.967 (19)) and temperature (from 35 °C to 14 °C (20)). In combination these studies revealed that *E. coli* O157:H7 appeared to lose, and then recover, culturability because of the effects of an abrupt downshift in a_w_; but not in temperature. Universal and distinct transcriptomic and proteomic responses to temperature and a_w_ downshift included an initial transient induction in the RpoE-dependent extracytoplasmic stress response and sustained expression of the RpoS-dependent general stress response pathway, as well as activation of the Rcs phosphorelay system involved in the biosynthesis of the exopolysaccharide colanic acid and downregulation of genes and proteins involved in chemotaxis and motility. These studies not only revealed the upregulation of genes and proteins that are part of an established adaptive response specific to cold temperature or hyperosmotic stress, but also revealed the unique upregulation of a number of genes and proteins not previously reported to be associated with survival and adaptation at low temperature and low osmolarity.

To gain a complete insight into the physiological response of exponentially growing *E. coli* O157:H7 Sakai to dynamic changes in conditions relevant to low temperature and water activity stress as occurs during carcass chilling, the present study was undertaken to investigate the growth kinetics and time-dependent changes in the transcriptome and proteome of exponential phase *E. coli* O157:H7 Sakai subjected to a sudden downshift in temperature and a_w_ from 35 °C a_w_ 0.993 to 14 °C a_w_ 0.967. The genome-wide expression response of *E. coli* was analyzed by both cDNA microarray (transcriptome response) and 2D-LC/MS/MS analysis (proteome response) as previously described (18–20), to characterize the potential mechanisms enabling this pathogen to adapt and/or survive under combined stresses.

## EXPERIMENTAL PROCEDURES

### 

#### 

##### Bacterial Strain and Preparation of inocula

The strain of *E. coli* O157:H7 Sakai (19, 20) used in this study was provided by Carlton Gyles (Guelph, Canada). A stock culture was maintained at −80 °C and was cultivated by plating onto brain-heart infusion (BHI) agar (Thermo Scientific, Waltham, MA, CM225) and incubating at 37 °C for 24 h. An isolated colony was inoculated into 25 ml of BHI broth and incubated at 37 °C for 20 h. The resulting broth culture was kept at 4 °C and used as a “working” culture within a week.

##### Preparation of Low a_w_ Broth

BHI broth containing a very high concentration of NaCl (a_w_ 0.760 ± 0.003) was prepared as described previously (18). The a_w_ of the NaCl-modified BHI broth was confirmed with an Aqualab CX-2 dew point instrument (Decagon Devices, Inc., Pullman, WA).

##### Abrupt Downshift in Both Temperature and Water Activity

An appropriate volume of the working culture was diluted 1:10^4^ into 25 ml of pre-warmed (35 °C) BHI broth. This “primary” culture was incubated at 35 °C with agitation (80 oscillations·min^−1^; Ratek Instruments, Boronia, Australia) and its growth was monitored turbidimetrically at 600 nm with a Spectronic 20 spectrophotometer (Bausch and Lomb, Rochester, NY). After achieving an OD_600_ of 0.1 ± 0.01 (*i.e.* the mid-exponential phase of growth, ∼10^7^ CFU.ml^−1^), a “secondary” culture was prepared by dilution of the primary culture by 1:10^2^ into 25 ml of prewarmed (35 °C) BHI broth. The secondary culture was incubated at 35 °C with agitation until it reached the mid-exponential growth phase (OD_600_ of 0.1 ± 0.01). The culture was then subjected to a sudden downshift in both temperature and water activity, by adding an appropriate volume of NaCl-concentrated BHI to yield a final a_w_ of 0.967 and by placing the culture at 14 °C in a shaking water bath.

##### Microbiological Analysis

Growth of *E. coli* was determined periodically by the viable count method, as described previously (18). The growth experiment was performed in quadruplicate. Construction of the growth curve and analysis of its kinetic parameters (*i.e.* generation time, GT^1^; lag time, LT; and adaptation time, AT) was undertaken according to Kocharunchitt *et al.* (2). Adaptation time (AT), *i.e.* the time taken for bacterial cells to resume exponential growth after the shift, was determined as cell death would mask the time when the lag period is actually resolved and would result in overestimation of the lag time (LT). Relative lag time (RLT) was calculated by dividing LT or AT with GT, to estimate the amount of *work* to be done by *E. coli* to adjust to the new conditions (21, 22). Where appropriate, statistical analysis was applied to assess differences in kinetic parameters using the Student's *t* test, at a confidence interval of 95% (*p* value = 0.05).

##### Preparation of Samples for Transcriptomic and Proteomic Analysis

For each experiment, *E. coli* cultures were subjected to an abrupt downshift from 35 °C a_w_ 0.993 to 14 °C a_w_ 0.967 as described above to prepare samples for cDNA microarray and 2D-LC/MS/MS analysis. Nine independent cultures (25 ml) were harvested either before an abrupt downshift in both temperature and a_w_ (*i.e.* control), or at 0 (*i.e.* immediately after the shift), 60, 250, 1605, 4070, 5700, 9900 or 18,565 min after the shift. All samples were subjected to the extraction protocol for RNA, and both soluble and membrane proteins, according to the methodology previously described (18). Table I describes the number of biological replicates performed for each time point in the transcriptomic and proteomic analysis.

**Table I:** The number of valid protein identifications and significantly differentially expressed genes and proteins after temperature and water activity downshift of E. coli O157:H7 Sakai from 35 °C a_w_ 0.993 to 14 °C a_w_ 0.967

Time point (min after the shift)	Number of biological replicates		Number of proteins*^b^*			Number of differentially expressed elements*^c^*		
	Transcriptome	Proteome*^a^*	Membrane fraction	Soluble fraction	Total fraction	Trancriptome (total)	Proteome (total)	Transcriptome *vs.* Proteome*^e^*
Control	3	6 (6)	300	1116	1162	NA*^d^*	NA	NA
0	3	2 (2)	266	416	507	6 (1)	97 (55)	1 (0)
60	3	2 (2)	258	489	566	79 (1)	83 (53)	3 (0)
250	3	2 (2)	139	291	341	277 (39)	44 (22)	7 (0)
1605	3	2 (2)	191	352	424	435 (346)	60 (41)	12 (7)
4070	3	2 (2)	366	531	581	537 (336)	144 (71)	68 (19)
5700	3	2 (2)	433	818	879	547 (386)	334 (120)	108 (35)
9900	3	2 (2)	366	611	687	528 (424)	223 (112)	96 (38)
18565	3	2 (2)	450	647	748	430 (404)	236 (132)	77 (42)

*^a^* Number of replicates performed for soluble (outside brackets) and membrane (within brackets) fractions of *E. coli*.

*^b^* Number of proteins identified in membrane and soluble fractions of the *E. coli* proteome that have passed the filtering criteria.

*^c^* Number of genes or proteins with increased (outside brackets) and decreased (within brackets) expression levels ≥ 1-log_2_ fold.

*^d^* NA; not applicable.

*^e^* Number of differentially expressed elements found in both the transcriptomic and proteomic analysis.

In the present study, the procedures of King *et al.* (3) and Kocharunchitt *et al.* (2) were carried out for cDNA microarray and 2D-LC/MS/MS analyses.

##### Microarray Data Analysis

Gene expression was analyzed using GeneSpring GX 10.0 software (Agilent Technologies, Palo Alto, CA). Array data was normalized using the Robust Multiarray Analysis (RMA) algorithm (23). Probesets with an intensity value in the lowest 20th percentile among all the intensity values were excluded. The remaining probesets, with intensity values between the 20th and 100th percentile, were used for downstream statistical analysis. Significance analysis was conducted using one-way analysis of variance (ANOVA) using the multiple correction testing method of Benjamini and Hochberg (24) with a *p* value cut-off of <0.01.

##### Analysis of MS/MS Data

MS/MS data obtained from each protein sample of membrane and soluble fractions were processed by the Computational Proteomics Analysis System (CPAS), a web-based system built on the LabKey Server (v9.1, released 02.04.2009) (25). The experimental mass spectra produced were subjected to a semitryptic search against the combined databases of *E. coli* O157:H7 Sakai (5318 entries in total) downloaded from the National Center for Biotechnology Information (NCBI, https://www.ncbi.nlm.nih.gov/, downloaded 25.11.2008) using X!Tandem v2007.07.01 (26). These databases included the *E. coli* O157:H7 Sakai database (5230 entries, NC_002695.fasta) and two *E. coli* O157:H7 Sakai plasmid databases, plasmid pO157 (85 entries, NC_002128.fasta) and plasmid pOSAK1 (three entries, NC_002127.fasta). The parameters for the database search were as follows: mass tolerance for precursor and fragment ions: 10 ppm and 0.5 Da, respectively; fixed modification: cysteine carbamidomethylation (+57 Da); and no variable modifications. The search results were then analyzed using the PeptideProphet and ProteinProphet algorithms from the Trans Proteomic Pipeline v3.4.2 (27, 28). All peptide and protein identifications were accepted at PeptideProphet and ProteinProphet of ≥0.9, corresponding to a theoretical error rate of ≤2% (29). To assess a false-positive discovery rate for each data set, the MS/MS spectra were searched against the target-reversed (decoy) database using the same search criteria as described above. The false-positive discovery rate of peptide identifications was then estimated by dividing the number of spectra matching decoy peptides with the total number of spectra, and appeared to be less than 4% for all data sets (supplemental Table S1).

All protein identifications that passed the above criteria were further assessed based on the confidence level of protein identifications across biological replicates at each time point. Their confidence level was determined as described previously (18). Specifically, proteins identified by more than one unique peptide in at least one of the replicates were considered to have a “high” confidence score. The “intermediate” confidence level was assigned to proteins with a single peptide hit that was detected in more than one replicate. “Low-confidence” proteins were considered to be those identified by a single unique peptide and found in only one replicate. Only protein identifications with a “high” and “intermediate” confidence level (referred to as having a “high confidence score”) were accepted for further analysis (see supplemental Tables S2–S10).

Following data filtering, all protein identifications with high confidence from membrane and soluble fractions of the same sample were combined to represent a “total” proteome of the corresponding sample. This combined approach was carried out to improve the reliability of the proteomic data. The R^2^ values, as determined by linear regression analysis indicated a relatively stronger linear correlation between spectral counts (SpCs, the number of MS/MS spectra) for all possible pair-wise comparisons of replicates of pooled fractions (average R^2^ = 0.92 ± 0.06) when compared with that of replicates of each fraction (average R^2^ = 0.74 ± 0.13, and 0.88 ± 0.13 for membrane and soluble fractions, respectively). Each of the total fractions across the biological replicates was then used to generate the final list of proteins considered to be representative of each time point of sampling.

##### Protein Abundance Ratio and Its Significance

Spectral count data generated by 2D-LC/MS/MS analysis were used as a semiquantitative measure of protein abundance (28, 30). The total abundance of each protein in a sample was obtained by pooling spectral counts detected for a given protein from membrane and soluble fractions of the same sample (*i.e.* the same biological replicate). Normalized spectral abundance factor (NSAF) was then applied to the pooled spectral count of each protein, according to Zybailov *et al.* (31). Briefly, the NSAF for a given protein is the pooled spectral count of the protein divided by its amino acid length (L), and normalized against the average SpC/L across all proteins in the corresponding data set. The NSAF values were then averaged across all biological replicates at each time point of sampling to obtain the representative values of NSAF.

Fold changes in protein abundance because of changes in environmental conditions were calculated as the log_2_ ratio of the average NSAF value in the sample over the average NSAF value of the control. To avoid errors during fold change calculation in cases where a zero value was obtained, a correction factor of 0.01 was added to each of the average NSAF values. It should be noted that the addition of the correction factor to the data did not have any significant impacts on the data, as indicated by the linear regression analysis of log_2_ ratios between the data with and without the correction factor (*i.e.* having an average slope of 1.12 ± 0.01 with R^2^ ≥ 0.98). Furthermore, differences in protein abundance were statistically analyzed using the beta-binomial test implemented in R (32). All proteins with a *p* value of <0.01 and at least 2-fold change were considered to be differentially abundant.

##### Transcriptomic and Proteomic Data Mining

Information on genes and proteins, including gene and protein names, ECs numbers (locus tag), GI numbers, NCBI Reference Sequence (RefSeq), protein sizes and molecular masses were obtained from the UniProt knowledgebase (http://www.uniprot.org/, accessed on 2.2.2011) and the National Center for Biotechnology Information (NCBI) database (https://www.ncbi.nlm.nih.gov/, accessed on 2.2.2011). Preliminary functions and properties of the proteins were based on the EcoCyc database collection (http://www.ecocyc.org/, accessed on 15.2.2011) and the KEGG database (http://www.genome.jp/kegg/pathway.html/, accessed on 25.2.2011). supplemental Table S11 contains an annotated list of all genes and proteins displaying a level of differential expression ≥ 1-log_2_ fold.

##### Predefined Set Enrichment Analysis

The T-profiler *t* test based method of Boorsma *et al.* (33) was performed on the log_2_ expression ratios, as previously described (19, 20), to determine changes in overall expression of a predefined set of proteins cold osmotic shock.

##### BiOLOG MicroPlate Assays

A study was conducted in parallel to the combined transcriptomic and proteomic study, to ascertain which substrates and products were utilized and produced by *E. coli* under the test conditions. Cultures were grown in duplicate and harvested at the time points previously described. PM1 and PM2 Biolog Phenotype Microarray plates were employed to test for carbon source utilization. Plates were inoculated according to the manufacturer's instructions, however the a_w_ of the inocula was adjusted with NaCl to an a_w_ of 0.967 and the inoculated Biolog plates were incubated at 14 °C. Microarray plates were read on a Wallac Victor 2 at an OD_595_ every 3 days for 30 days and a final reading was taken at 40 days. Positive well readings were those in which the OD_595_ was greater than twice that of the negative control. A carbon source was deemed as being utilized only if both biological replicates returned a positive reading for that carbon source.

##### Validation of Microarray Data by qRT-PCR

Microarray results were validated using quantitative real-time reverse transcription-PCR (qRT-PCR) as described previously (20). The siroheme synthase gene (*cysG/ECs4219*) was included for normalization within samples (34). Forward and reverse PCR primers for eight genes were designed using Primer3 software (35) and are listed in supplemental Table S12. One microgram of purified total RNA was reverse transcribed using the iScript cDNA synthesis kit (Bio-Rad, Hercules, CA). Real-time PCR was performed on 10-fold dilutions (from 10^−2^ to 10^−4^) of the template cDNA. qRT-PCR mixes contained a total volume of 20 μl consisting of 10 μl IQ SYBR green supermix (Bio-Rad), 1.6 μl diluted cDNA, 0.4 μl each forward and reverse primer (25 μm stock), and 7.6 μl nuclease-free water (Ambion, Austin, TX). All Real-time PCR reactions were performed on the LightCycler 480 (Roche Applied Science, West Grove, PA) under the cycling conditions of: 95 °C for 3 min, 45 consecutive cycles consisting of 95 °C for 10 s and 60 °C for 30 s, and 72 °C for 30 s. Melting curve analysis (55 to 95 °C, increment of 0.11 °C/sec) was performed to ensure PCR specificity. The method described by Pfaffl (36) was employed to determine the expression fold changes of the target gene in cultures at each time point postcombined water activity and temperature downshift, compared with the reference culture.

##### Accession Numbers

Transcriptomic data was deposited at ArrayExpress (http://www.ebi.ac.uk/arrayexpress/) under accession number E-MTAB-3969. The proteomic data was deposited to the ProteomeXchange Consortium via the PRIDE (37) partner repository under accession numbers PXD004803. The peptide identification results can be viewed using MS-Viewer (http://prospector2.ucsf.edu/prospector/cgi-bin/msform.cgi?form=msviewer) (38), using the search keys listed in supplemental Table S1.

## RESULTS AND DISCUSSION

### 

#### 

##### Growth of E. coli O157:H7 Sakai Upon Cold Osmotic Shock

**Fig. 1.**
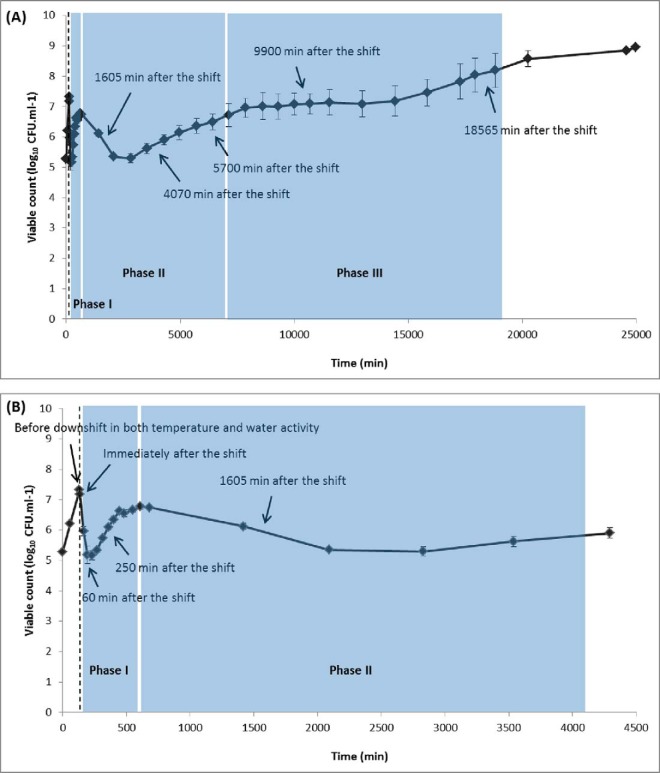
**Growth response and sampling scheme for analysis of the cellular response of *E. coli* O157:H7 Sakai to a sudden downshift in temperature and water activity from 35 °C a_w_ 0.993 to 14 °C a_w_ 0.967.** The time at which a downshift in temperature and a_w_ was applied is indicated by a dotted line. Block solid arrows indicate the sampling points for cDNA microarray and 2D-LC-MS/MS analysis. Data points represent means ± standard deviations of at least two independent replicates. Panel (*A*) shows all available data and panel (*B*) shows all data on a reduced time scale.

Exponentially growing *E. coli* cultures were exposed to a sudden downshift in temperature and water activity from 35 °C a_w_ 0.993 to 14 °C a_w_ 0.967. This combined downshift induced a complex pattern of microbial behavior, resulting in a growth curve that could be divided into three successive phases (Fig. 1). In the first phase cell numbers initially declined and then increased rapidly. In the second phase cell numbers decreased again and a period of “exponential-like” growth followed. In the third phase the population number remained at a similar level to the starting population until “true exponential” growth was established. Kinetic parameters were determined as described previously (19–22, 39) for each phase of the growth curve (Table IIG).

**Table II:** Summary on the growth response of exponential phase E. coli O157:H7 Sakai to a sudden downshift in temperature and water activity from 35 °C a_w_ 0.993 to 14 °C a_w_ 0.967, as determined by viable count

Trial	Starting number (CFU.ml^−1^)*^a^*	Phase I			Phase II			Phase III				
		GT (min)*^b^*	AT (min)*^c^*	RLT (AT/GT)	GT (min)	AT (min)	RLT (AT/GT)	GT (min)	AT (min)	LT (min)*^d^*	RLT	
											AT/GT	LT/GT
1	7.36										13.66	15.86
2	7.30	37	72	1.95	872	2100	2.41	1088	13,740	13,820	12.63	12.70
3	7.53	86	78	0.91	922	2340	2.54	931	14,220	16,957	15.27	18.21
4	7.48	66	84	1.27	1015	2220	2.19	1044	13,800	17,191	13.22	16.47
Mean	7.42	57	75	1.44	963	2220	2.31	1028	14,025	16,154	13.69	15.81
S.D.	0.11	23	8	0.45	80	98	0.19	68	299	1,572	1.13	2.30

*^a^* The number of *E. coli* at which a downshift in temperature and a_w_ was applied.

*^b^* Generation time (GT) is determined by linear regression for each phase.

*^c^* Adaptation time (AT) is estimated visually for each phase, as the time taken for cells to resume the exponential growth after applying the shift.

*^d^* Lag time (LT) is calculated by linear regression as the time taken to increase above starting numbers.

The GT for phase I was significantly shorter (*p* value <0.05) than that of phase II and III, whereas there was no significant difference in the GT between phase II and III (Table II). Both AT and RLT were significantly different (*p* value <0.05) between phases and increased in magnitude with increasing phase number. Furthermore, the LT was significantly longer (*p* value <0.05) than that of the AT for phase III. This observation aligns with our previous study in which cell injury or death caused by osmotic stress was thought to mask the time at which the actual lag period was resolved, leading to an overestimation of the lag time (19). However, the estimates of RLT for phase III; as determined by dividing LT or AT with GT, did not show a statistically significant difference.

The initial decline in cell numbers during phase I suggests a loss in cell culturability on enumeration media as a result of injury (Fig. 1). This loss of culturability was followed by a period of cell recovery at a rate (*i.e.* GT of phase I) significantly faster (*p* value <0.05) than those of phases II and III (Table II). In support of these results, Mellefont *et al.* (14) demonstrated a transient loss of the ability of *E. coli* to produce colonies on enumeration plates when chilling and desiccation profiles were applied simultaneously. Our previous studies also reported that hyperomostic shock alone (from a_w_ 0.993 to a_w_ 0.967) could result in the injury and recovery phenomenon (19), however this was not the case for cold shock alone (from 35 °C to 14 °C) (20). Taken together, the results of the current and previous studies (19, 20) indicate that the apparent loss and subsequent recovery of culturability upon exposure to combined cold and osmotic shock, is because of the effects of an abrupt downshift in a_w_ and not temperature. This is also consistent with the results of Kocharunchitt *et al.* (16), indicating that water activity stress has a greater effect on the physiology of *E. coli* than cold stress.

There was no significant difference between the GTs observed in phase II and III (Table II), indicating that exponential growth may have already resumed from phase II onward. However, the basis of the subsequent period in which bacterial numbers remained constant during phase III (Fig. 1) is still unclear. Further investigation by culture-independent methods may further our understanding of the growth response of *E. coli* to cold osmotic shock. For example, insight would be provided though the examination of the ultrastructure of the cells as they appear to undergo rapid division and recover viability.

##### Molecular Response of E. coli O157:H7 Sakai to Temperature and Osmotic Downshift

Time-dependent changes in the gene expression and protein abundance profiles of *E. coli* in response to temperature and a_w_ downshifts were investigated by microarray and 2D-LC/MS/MS analyses. The proteomic analysis identified a number of proteins with high confidence in membrane and soluble fractions of *E. coli* at each time point (Table I and supplemental Tables S2–S10). A number of genes and proteins were observed to be differentially expressed by *E. coli* when subjected to environmental changes (Table I). supplemental Table S11 presents a complete and annotated list of these genes and proteins with a comparison to previously published studies.

##### Comparison of Transcriptome and Proteome

The level of correlation between transcriptomic and proteomic data was determined by calculating the percentage of differentially expressed genes and proteins matched at each time point. The level of correlation appeared to be low, with an average of only 8.5% ± 7.4% of genes and proteins matched across all time points. To validate the results of the microarray study, qRT-PCR was used to determine the expression level of several selected candidate genes. The results of the qRT-PCR experiments confirmed the results of the microarray study (supplemental Table S13). The apparent low level of correlation between the transcriptomic and proteomic data is consistent with previous studies, supporting the idea that different control mechanisms are present at the transcriptomic and proteomic levels, and that a combination of mRNA and protein expression patterns are required to fully develop an understanding of the functional architecture of genomes and gene networks (18–20, 40).

**Fig. 2.**
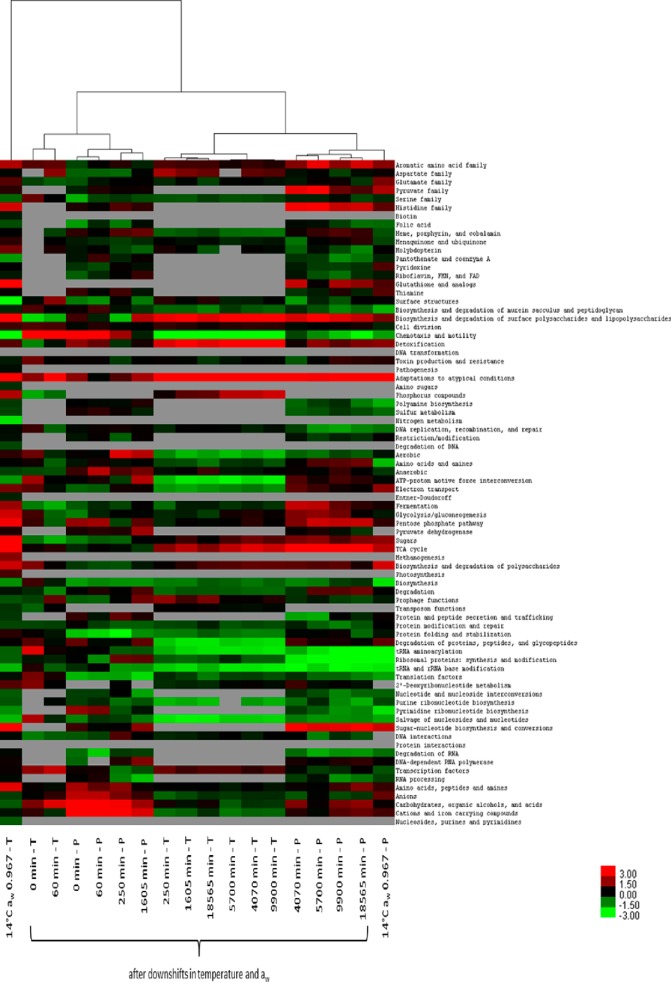
**Hierarchical clustering analysis of combined transcriptomic and proteomic data.** The heat map represents the *T*-values calculated for transcriptomic (T) and proteomic (P) profiles based on JCVI CMR functional categories (row) and is linked by a dendrogram representing clustering of these profiles obtained from *E. coli* O157:H7 Sakai during exposure to simultaneous downshifts in temperature and a_w_ and those during exponential growth at 14 °C aw 0.967 (18) (top). The color code is as follows: red indicates positive T-values; green indicates negative T-values; and gray indicates not determined.

To further determine the similarity between gene and protein profiles across different time points, hierarchical clustering analysis was performed on the T-values obtained in the present study (*i.e.* both transcriptomic and proteomic data) and that of Kocharunchitt *et al.* (18) on the transcriptomic and proteomic responses of *E. coli* during exponential growth at the same stress conditions (14 °C a_w_ 0.967). It was found that gene and protein profiles could be grouped into three major clusters (Fig. 2). The first cluster consists of the gene profiles within the first 60 min following downshifts in temperature and a_w_ and the protein profiles within the first 1605 min, whereas the second cluster is comprised of the gene profiles from 250 min onwards, and the protein profiles from 4070 min onwards and that of steady-state growth at 14 °C a_w_ 0.967. The last cluster only contains the gene profile of steady-state growth at 14 °C a_w_ 0.967. These findings agree well with previous studies (19, 41), reporting that the transcriptomic data at a given time point was a reflection of subsequent, rather than concomitant proteomic data, providing a valid explanation for the low level of correlation between gene and protein profiles observed here (*see* above). It also should be noted that although the gene profile of *E. coli* during steady-state growth at 14 °C a_w_ 0.967 did not cluster with any other profiles, the apparent cluster of the protein profiles from 4070 min onwards and that of *E. coli* grown under the condition of 14 °C a_w_ 0.967 suggests that that balanced growth typical of growth at 14 °C a_w_ 0.967 has been established from 4070 min onward. This supports the hypothesis derived from the growth study, that *E. coli* might have already resumed growth from phase II of the growth curve onward. Taken together, it could be hypothesized that there were only two physiological states of *E. coli* during exposure to combined cold and water activity stresses, despite the complex pattern of their growth behavior observed (*see* above). These were growth arrest or adaptation phase, and regrowth phase. However, the microbial physiology of the subsequent period, in which bacterial numbers were unchanged, is still unclear (*i.e.* during phase III).

##### Time-dependent Response

To investigate the expression pattern of genes and proteins under conditions of combined cold and osmotic stress, the present study employed the K-means clustering algorithm together with principal-component analysis to analyze genes and proteins with an altered expression level at one or more time points. Based on the principal-component analysis, it was evident that the gene and protein profiles could be respectively explained by three and five principal components, accounting for ∼95% of the variance (data not shown). This means that three major independent features were required to categorise the time-dependent expression level of each gene, whereas there were five major features for proteomic profiles (42). The apparent differences in the characteristics of gene expression and protein abundance reflect the complexity of the mechanisms of adaptation used by *E. coli* when exposed to combined cold and water activity stresses. Of particular note is that *E. coli* cells appeared to primarily regulate their protein abundance (possibly through the processes of translation and post-translational modification) to quickly adapt or respond to such stresses (as discussed in more detail below). In keeping with this, previous studies have reported a similar observation for the transcriptome and proteome of *E. coli* during exposure to hyperosmotic shift (19) and *Saccharomyces cerevisiae* during alcoholic fermentation (41).

**Fig. 3.**
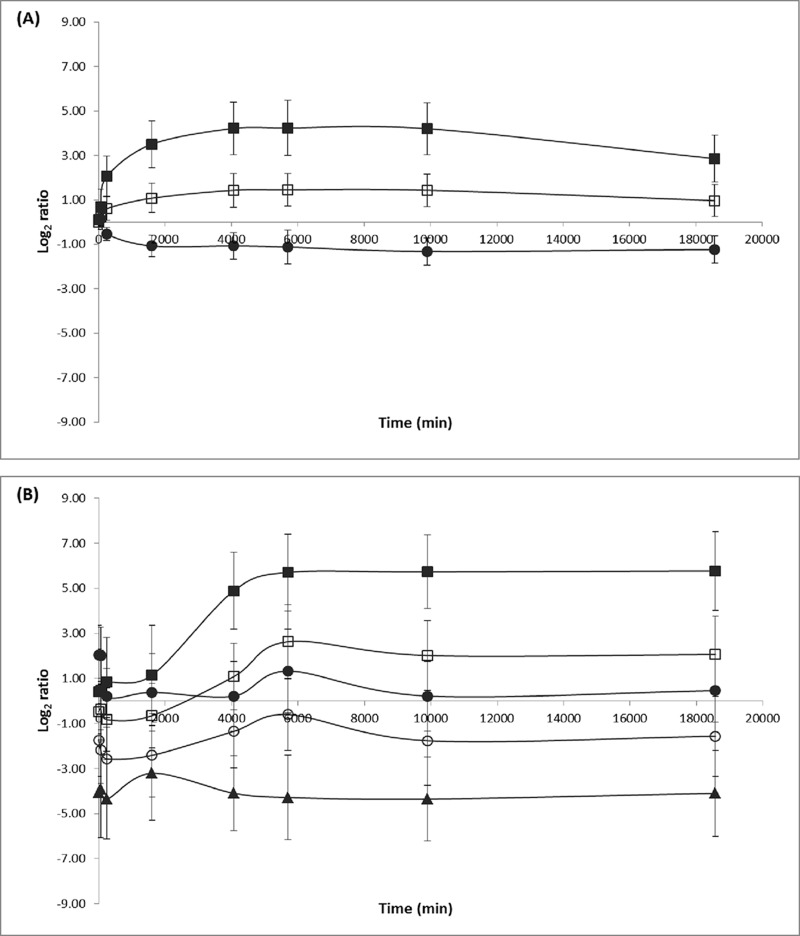
**Changes in the average expression levels of genes and proteins in each cluster over the course of the experiments, as determined by K-means clustering analysis.** K-means clustering assigned genes and proteins with similar expression characteristics into three (Clusters I (■), II (□) and III (●)) and five (Clusters A (■), B (▴), C (○), D (□) and E (●)) different clusters, respectively.

Genes and proteins with similar expression characteristics were assigned into three (Clusters I–III) and five (Clusters A–E) different clusters by the K-means clustering analysis, respectively. Fig. 3 describes the average expression of genes and proteins in each cluster over the course of the experiment. The complete list of genes and proteins in each cluster is given in supplemental Table S11. Because of the complexity of the transcriptomic and proteomic data and their low levels of correlation, the data from each approach will be described and discussed separately. However, the biological significances, where the common trends were observed, will be highlighted.

##### Gene Cluster I

The first gene cluster was characterized by 178 genes that displayed an increase in expression from 250 min after the stresses were imposed (Fig. 3*A*). This pattern of expression corresponds well with the period at which *E. coli* had recovered from the injury as a result of the stresses. This suggests that proteins encoded by those genes in this cluster might help *E. coli* to adapt and survive under the stress conditions. In keeping with this, cluster I includes the master stress regulator *rpoS* (*ECs3595*) and the extracytoplasmic stress response regulator *rpoE* (*ECs3439*). The T-profiler analysis of the transcriptomic study also revealed upregulation of the RpoS regulon from 250 min onwards, and the RpoE regulon from 60 min onwards (supplemental Table S14). Cluster I contains a number of other stress responsive genes involved in regulation (*osmE*/*ECs2445*, *rseC*/*ECs3436*, *soxS*/*ECs5044*, *ybaY*/*ECs0507*), the osmotic stress response (*osmB*/*ECs1856*, *osmC*/*ECs2086*), cold shock response (*cspG*/*ECs1145*), oxidative stress response (*katE*/*ECs2438*), DNA protection (*dps*/*ECs0890*), synthesis of trehalose (*otsAB*), and control of the intracellular level of trehalose (*treF*/*ECs4399*). This correlates with the T-profiler analysis, which revealed upregulation of genes from 60 mins onwards, involved in adaptations to atypical conditions (supplemental Table S14). Within this functional category, the cold shock protein encoded by *cspG*/*ECs1145* was among the most highly expressed genes at every time point displaying up to a 6.66-log_2_ fold increase in expression (supplemental Table S11).

The T-profiler analysis of the transcriptomic data revealed the upregulation of the Rcs regulon from 250 min onwards (Supplemental Table S14) and cluster I includes a number of genes involved in the biosynthesis of colanic acid (*wcaM*/*ECs2848*, *wcaL*/*ECs2849*, *wcaK*/*ECs2850*, *ECs2851*, *wcaJ*/*ECs2852*, *wcaI*/*ECs2855*, *wcaG*/*ECs2857*, *gmd*/*ECs2858*, *wcaF*/*ECs2859*, *wcaE*/*ECs2860*, *wcaC*/*ECs2862*, *wcaB*/*ECs2863*, *wcaA*/*ECs2864*, *wzc*/*ECs2865,* and *yjbF*/*ECs5010*).

Cluster I also contained a number of genes involved in energy metabolism, including genes of the TCA cycle (*gltA*/*ECs0745*, *acnA*/*ECs1849*, *fumC*/*ECs2317*). The T-profiler analysis of the transcriptomic data also revealed upregulation of the TCA cycle from 1605 min onwards (supplemental Table S14). Genes involved in the pentose phosphate pathway, namely *talA*/*ECs3326* and *tktB*/*ECs3327*, were also upregulated in keeping with the fact that their transcription is positively regulated by RpoS (43).

Over 40% of the genes in cluster I encode hypothetical proteins (supplemental Table S11), indicating that further work is required to further characterize the physiological response of *E. coli* to combined osmotic and chill temperature stress.

##### Gene Cluster II

The 447 genes in cluster II displayed a similar expression profile to cluster I, although their magnitude of induction was smaller (Fig. 3*A*). Cluster II also contained genes of the RpoS regulon including those involved in acid resistance (*gadB*/*ECs2098*), cell filamentation (*fic*/*ECs4212*), and encoding hypothetical proteins (*ECs0383*, *ECs0886*, *ycaC*/*ECs0982*, *ECs1692*, *ECs2695*, *ECs2785*, *ECs2888*, *ECs3588*, *ECs4112*, *yhfG*/*ECs4213*, *ECs4323*, *ysgA*/*ECs4760*, *ECs5089*).

Cluster II contains genes involved in fatty acid and phospholipid metabolism (*fadA*/*ECs4773*, *fadI*/*ECs3225*). Genes involved in energy metabolism were upregulated, including those involved in glycogen degradation (*ECs0453*), the TCA cycle (*sdhC*/*ECs0746*, *sdhD*/*ECs0747*, *sucC*/*ECs0753*, *ECs4933*), mixed acid fermentation (*aldA*/*ECs2021*), glycolysis (*ECs2022*), trehalose biosynthesis, and glycogen degradation (*glgX*/*ECs4276*). However, as stated previously, the T-profiler analysis of the transcriptomic data only revealed positive regulation of genes of the TCA cycle (supplemental Table S14). Genes involved in central intermediary metabolism were upregulated, including those involved in glycine betaine biosynthesis (*betB*/*ECs0358*) and glutamate dependent acid resistance (*gadB*/*ECs2098*). Genes involved in histidine biosynthesis were upregulated (*hisD*/*ECs2821*, *hisC*/*ECs2822*, *hisH*/*ECs2824*), as well as genes involved in isoleucine and valine biosynthesis (*ilvB*/*ECs4612*, *ilvC*/*ECs4708*).

Cluster II contains a number of genes encoding transport and binding proteins, including those involved in the uptake of glycine betaine/proline (*proV*/*ECs3540*, *proW*/*ECs3541*), transport proteins for charged solutes such as glutamate (*ECs0692*, *ECs0693*), a putrescine transporter ATP-binding subunit (*ECs0935*) and histidine transport system permease protein (*ECs3192*).

A number of genes involved in DNA metabolism were upregulated, including a DNA damage-inducible gene (*dinJ*/*ECs0253*), a UV-induced DNA repair gene (*ECs0733*), a nucleotide excision repair endonuclease (*ECs2447*), the SOS response to DNA damage (*recA*/*ECs3556*), subunits of the UvrABC nucleotide excision repair complex (*uvrA*/*ECs5040*), and the response regulator of RpoS (*ECs1737*). Cluster II also contains *clpA*/*ECs0968*, which catalyzes the removal of both misfolded and properly folded proteins. We also observed upregulation of the outer membrane lipoprotein involved in stationary-phase cell survival (*nlpD*/*ECs3596*).

As for cluster I, over 40% of the genes within cluster II encode hypothetical proteins or have no known function (supplemental Table S11). This reinforces the need for further characterization of the physiological response of *E. coli* to combined osmotic and chill temperature stress.

##### Gene Cluster III

This cluster consists of 541 genes that were downregulated from 250 min after exposure to combined cold and osmotic stresses (Fig. 3*A*). This indicates that proteins encoded by genes with this expression pattern might not be required for cells to resume growth and survive under the stress conditions. A number of genes within cluster III are involved in chemotaxis and motility (*ECs1450*, *ECs1451*, *flgC*/*ECs1452*, *ECs1454*, *ECs1455*, *ECs1456*, *ECs1457*, *flgI*/*ECs1458*, *ECs1459*, *ECs2588*, *ECs2589*, *ECs2601*, *ECs2661*, *fliE*/*ECs2676*, *fliF*/*ECs2677*, *ECs2678*, *ECs2679*, *ECs2680*, *ECs2681*, *ECs2682*, *ECs2683*, *ECs2684*, *ECs2685*, *ECs2686*, *ECs2687*, *ECs2688*, *ECs2689*, *ECs5315*). The regulator of flagellar biosynthesis (*flhD*/*ECs2602*) is also present in this cluster. These results align with the results of the T-profiler analysis, which revealed a significant decrease in the overall expression of genes involved in chemotaxis and motility from 250 min of the shift onwards (supplemental Table S14). Consistent with these, Kocharunchitt *et al.* (18) has suggested that chemotaxis and motility systems are the most dispensable functions during steady-state growth under combined cold and osmotic stress (14 °C aw 0.967). It is also worthwhile noting that the apparent downregulation of genes involved in chemotaxis and motility, is in line with the negative regulation of genes encoding flagella by RpoS (44) and Rcs (45).

Genes involved in energy metabolism were also among those genes downregulated in cluster III. Significant downregulation was observed for genes involved in aerobic respiration (*ECs3161*, *ECs3162*, *ECs3163*, *ECs3164*, *ECs3166*, *ECs3168*, *ECs3169*, *nuoC*/*ECs3170*, *nuoB*/*ECs3171*), oxidative phosphorylation (*cydA*/*ECs0768*, *ECs0769*, *ECs3161*, *ECs3162*, *ECs3163*, *ECs3164*, *ECs3166*, *ECs3168*, *ECs3169, nuoC*/*ECs3170*, *nuoB*/*ECs3171*, *ECs3172*, *atpD*/*ECs4674*, *atpG*/*ECs4675*, *atpH*/*ECs4677*, *atpF*/*ECs4678*, *ECs4679*, *ECs4680,* and *ppa*/*ECs5204*), ATP-proton motive force interconversions (*ECs4680*, *ECs4338*, *atpG*/*ECs4675*, *atpH*/*ECs4677*, *atpF*/*ECs4678*, *atpD*/*ECs4674*, *ECs2680*, *ECs4679*), and electron transport (*ECs0769*, *ECs0988*, *nfnB*/*ECs0616*, *ECs2308*, *nuoB*/*ECs3171*, *ECs3166*, *cydA*/*ECs0768*, *ECs3162*,*ECs3168*, *ECs1031*, *ycbY*/*ECs1032*, *nuoC*/*ECs3170*). These results correlate with the T-profiler analysis of the transcriptomic data (supplemental Table S14), with significant downregulation observed for genes involved in aerobic respiration (1605 min onward), oxidative phosphorylation (250 min onward), ATP-proton motive force interconversion (250 min onward), and electron transport (250 min onward). This cluster also contained genes involved in nitrogen metabolism (*ECs1321*, *ECs1322*, *ECs1325*, *ECs1327*), sulfur metabolism (*ECs3605*, *cysI*/*ECs3618*, *cysJ*/*ECs3619*), and folate biosynthesis (*folA*/*ECs0051*, *moaC*/*ECs0861*, *moaD*/*ECs0862*, *ECs2521*, *folB*/*ECs3941*). However, the T-profiler analysis of the transcriptomic data did not display a significant trend in the expression of these genes (supplemental Table S14).

Cluster III includes a number of genes that play an essential role in cell division (*minE*/*ECs1668*, *minD*/*ECs1669*, *ECs3432*). However, this trend was not observed in the T-profiler analysis of the transcriptomic data (supplemental Table S14). Cluster III also contains genes involved in the salvage of nucleosides and nucleotides, pyrimidine ribonucleotide biosynthesis, and purine ribonucleotide biosynthesis. This pattern of expression was also shown to be significant in the T-profiler analysis, with the transcriptomic data revealing a significant downregulation in genes involved in the salvage of nucleosides and nucleotides from 250 min onwards (supplemental Table S14). Cluster III contained a number of genes involved in DNA repair (*sbcD*/*ECs0448*, *xthA*/*ECs2455*, *mutS*/*ECs3589*, *radA*/*ECs5347*, *uvrC*/*ECs2651*) and metabolism (*hepA*/*ECs0063*). The downregulation of *hepA*/*ECs0063* is unexpected, as HepA is a transcriptional regulator that activates transcription by stimulating RNA polymerase (RNAP) recycling in case of stress conditions such as supercoiled DNA or high salt concentrations. In addition, around 5% of the genes within cluster III are involved in protein synthesis. Cluster III also contained a number of genes involved in protein folding and stabilization (*ECs0587*, *ECs2878*, *ECs3084*, *ECs3584*, *ECs3585*, *ECs5123*) and protein modification and repair (*ECs0970*, *ECs3582*, *ECs5345*).

Surprisingly, cluster III also contains a number of genes with established roles in the osmotic stress response, such as cardiolipin synthetase (*ECs1749*). Increasing cardiolipin content is a key player in bacterial adaptation to osmotic stress. In addition, we observed downregulation of the transcriptional regulator of the *kdp* operon involved in potassium transport (*kdpE*/*ECs0722*), a component of the KDP system (*ECs0724*) and the osmolarity response regulator (*ompR*/*ECs4247*). Although cold and osmotic stress induce a defense response mechanism against oxidative stress that includes the induction of elements involved in iron acquisition, this cluster also included genes involved in iron acquisition (*fepA*/*ECs0623*, *fiu*/*ECs0883*).

##### Protein Cluster A

The first protein cluster represents 94 proteins that exhibited an increase in abundance level at 4070 min after simultaneous cold and osmotic shifts and maintained their level of upregulation throughout the stress (Fig. 3*B*). This abundance pattern corresponds well with the period at which *E. coli* had resumed exponential growth after applying the shifts (*i.e.* from phase II of the growth curve onward; *see* above). This indicates that the proteins in this cluster might be part of adaptive strategies and responses to mediate growth and/or survival of *E. coli* under combined cold and osmotic stress. It also should be noted that the expression characteristic of proteins in cluster A was similar to that of gene cluster I (*see* above and Fig. 3). This is despite the time delay between the expression of genes in cluster I; which displayed an increase in expression 250 min after the stresses were imposed, and proteins in cluster A; which displayed an increase in abundance after 4070 min. The apparent delay in time of the gene expression and protein abundance changes reinforces the previous idea that the transcriptomic data at a given time point was a reflection of subsequent, rather than concomitant proteomic data (19, 41).

Cluster A is mainly composed of proteins with functions related to a stress response. These included chaperone proteins (IbpA/ECs4627 and Spy/ECs2449), which have functions in facilitating protein folding, and preventing aggregation of misfolded proteins; PspA/ECs1881, which is responsible for maintaining the proton motive force under stress conditions; YncC/ECs2054 that regulates biofilm formation and mucoidity; YtfE/ECs5187, which is involved in the repair of damaged iron-sulfur clusters under nitrosative and oxidative stress conditions; and DkgA/ECs3896 that is involved in the detoxification of methylglyoxal (a toxic compound produced during glycolysis, fatty acid metabolism, and protein metabolism).

One of the major cold shock proteins (CspG/ECs1145), whose gene is also present in cluster I (*see* above), exhibited an increase in abundance during growth under combined cold and osmotic stress. This cold shock protein is thought to have overlapping functions with the well-studied cold shock protein CspA, as an RNA chaperone. Increased production of CspG/ECs1145 has also been reported to be essential for bacterial cells to resume growth at low temperature (46, 47). However, the abundance pattern of CspG/ECs1145 observed here contradicts previous proteomic studies using two-dimensional difference gel electrophoresis (2D-DIGE), which demonstrated that CspG is upregulated during acclimation phase upon cold shock, and subsequently downregulated when cells have become cold-adapted (46, 48). This was despite the recent findings that have indicated an upregulation of *cspG*/CspG in *E. coli* cells at which growth was resumed after a temperature downshift (20). It also should be noted that the present study found a transient decrease in abundance of CspA/ECs4441 following simultaneous cold and osmotic shifts (*i.e.* Cluster C; *see* below). These observations, therefore, suggest that CspG may play a more important and specific role than CspA in response to the stress conditions used in this study. In support of this, Yamanaka (47) reported that CspG is induced within a narrower range of low temperature than CspA.

*E. coli* responded to combined cold and osmotic stress by upregulating the master stress regulator RpoS/ECs3595, and a group of proteins whose transcription is positively regulated by RpoS (43, 44, 49), as indicated by their presence in cluster A. This observation agrees well with gene cluster I (*see* above), and is consistent with the T-profiler analysis, revealing a significant increase in the overall abundance of proteins involved in the RpoS regulon from 250 min after application of the combined cold and osmotic stress onward (supplemental Table S14). More specifically, the majority of these RpoS-dependent proteins are involved in osmotic adaptation (18, 50–52). These included osmotically inducible proteins (OsmB/ECs1856, OsmC/ECs2086, OsmE/ECs2445, OsmY/ECs5334); Dps/ECs0890, which is responsible for the protection of DNA; OtsA/ECs2604 and OstB/ECs2605, which are involved in the *de novo* synthesis of trehalose; TreF/ECs4399, which controls the intracellular level of trehalose; YhbO/ECs4034, which regulates RNA/protein interaction, signal transduction, thiamine biosynthesis and proteases; and a group of proteins with undefined functions (ElaB/ECs3154, YciE/ECs1829, YciF/ECs1830, YgaM/ECs3533, and YjbJ/ECs5028). Other proteins of the RpoS regulon in this cluster were also identified, and appear to be associated with the response to oxidative damage, such as KatE/ECs2438, SufA/ECs2391, SufC/ECs2389, and SufS/ECs2387. In keeping with this, Kocharunchitt *et al.* (18) has demonstrated activation of the master stress regulator RpoS during exponential growth of *E. coli* under the same stress condition (14 °C a_w_ 0.967). The present findings also align with previous studies, reporting that the general response network established by the RpoS regulon typically provides cross-protection against diverse stress conditions (18, 43, 44, 49). Furthermore, it has previously been demonstrated that prolonged exposure of *E. coli* cells to cold stress (14 °C) resulted in downregulation of several elements involved in the response to oxidative stress (20), whereas expression of these elements was induced during growth at hyperosmolarity (a_w_ 0.967) (19). This has led to the suggestion that the increase in abundance of oxidative stress-responsive proteins observed here may be because of the effects of osmotic stress rather than cold stress. However, Kocharunchitt *et al.* (18) reported upregulation of several genes involved in oxidative stress resistance in *E. coli* cells grown under the conditions of low temperature (14 °C a_w_ 0.985) and a_w_ stress (25 °C a_w_ 0.967).

Another group of important proteins in cluster A, most of whose corresponding genes are present in cluster I (*see* above), are those involved in the Rcs phosphorelay system (*i.e.* WcaC/ECs2862, WcaG/ECs2857, WcaI/ECs2855, WcaK/ECs2850, WcaL/ECs2849, Wza/ECs2867, and Wzc/ECs2865). This system is known to regulate the biosynthesis of the exopolysaccharide colanic acid (53). Consistent with these observations, Kocharunchitt *et al.* (18) demonstrated that *E. coli* strongly upregulated Rcs-dependent elements and produced a high level of colanic acid during steady-state growth under conditions of combined cold and osmotic stress (14 °C a_w_ 0.967). Colanic acid has been reported to play an important role in protecting bacterial cells from a variety of environmental stresses, including both low temperature and osmotic stress (54–56), and has been shown to be essential for biofilm formation (57). Despite this, the mechanism of stress tolerance conferred by colanic acid remains to be elucidated. Several studies have also suggested that the properties of colanic acid, *i.e.* to create a physical barrier with a strong negative charge on the outer cell surface, might be one of the contributing factors that mediate the tolerance of cells to stressful conditions (55, 56, 58). However, it is worth noting that Kocharunchitt *et al.* (18) found that colanic acid was not required for growth and survival under combined cold osmotic stress. Further research, therefore, should be conducted to elucidate the physiological function of colanic acid during exposure of bacterial cells to stress conditions.

##### Protein Cluster B

Cluster B consists of 90 proteins that were downregulated during the whole period of exposure to combined cold and osmotic stress (Fig. 3*B*). It is suggested that proteins with this abundance pattern may not be required for cells to resume growth and survive under these stress conditions. In addition, a decrease in synthesis of these proteins may prevent an inappropriate use of cellular energy. Cluster B consists of a wide range of proteins with diverse functions (supplemental Table S11). Notably, this cluster includes a group of proteins (Rnc/ECs3433 and Rnd/ECs2513), that are responsible for RNA degradation; TrmA/ECs4896, TrmB/ECs3836, and TrmJ/ECs3398, which have functions in tRNA and rRNA base modification; SdaB/ECs3657 that is a key enzyme in the pathway of serine degradation; and WbdO/ECs2843 and WbdQ/ECs2837, which are putative enzymes involved in the biosynthesis of lipopolysaccharide.

##### Protein Cluster C

Cluster C is composed of 183 proteins whose abundance was transiently reduced within the first 1605 min of combined cold and osmotic stress (Fig. 3*B*). The abundance characteristics of these proteins is consistent with the period at which growth arrest was observed upon simultaneous cold and osmotic shifts (*see* above). This indicates that the functions of these proteins may contribute to the prolonged growth arrest of *E. coli* after the shifts. Cluster C also contained a number of proteins involved in defense mechanisms against oxidative damage, including alkylhydroperoxide reductase (AhpC/ECs0644 and AhpF/ECs0645), Hydroperoxidase I (KatG/ECs4871), and lipid hydroperoxide peroxidase (Tpx/ECs1903). This finding is consistent with the study of Mackey and Derrick (59) demonstrating that *E. coli* was sensitized to oxidative stress upon cold shock. It has also been shown that several oxidative stress-responsive elements were downregulated during growth arrest upon a temperature downshift (from 35 °C to 14 °C) (20), whereas no obvious trend of response was observed for expression of these elements following hyperosmotic shock (from a_w_ 0.993 to a_w_ 0.967) (19). This indicates that the apparent downregulation of proteins involved in the oxidative stress response may be related to the effects of cold shock rather than osmotic shock.

Exposure of *E. coli* to sudden downshifts in temperature and a_w_ resulted in a transient downregulation of several chaperones (ClpX/ECs0492, DnaK/ECs0014, GrpE/ECs3476, and HtpG/ECs0526), as indicated by their presence in cluster C. All of these chaperones are of the RpoH regulon, which is responsible for the control of protein misfolding in the cytoplasm (60, 61). Accordingly, the T-profiler analysis revealed a significant decrease in the overall abundance of proteins involved in protein folding and stabilization within the first 250 min of the stress and those proteins involved in the RpoH regulon at 250 min (Fig. 3*B*). This was despite the observation that RpoH/ECs4310 did not exhibit a significant change in abundance. The apparent downregulation of chaperone proteins during growth arrest indicates that *E. coli* may lose efficiency in repairing misfolded proteins and in facilitating the proper folding of newly synthesized proteins following combined cold and osmotic shifts. In keeping with this, Strocchi *et al.* (62) report that the growth arrest of *E. coli* upon cold shock may be associated with inactivation of the chaperone system. It has also been shown previously that a sudden downshift in temperature resulted in downregulation of several elements involved in protein folding machinery (20, 63). Additionally, it is worthwhile noting that other chaperone proteins of the RpoH regulon were observed in this study. Many of these (DnaJ/ECs0015, GroEL/ECs5124 and HtpX/ECs2539) appeared to be upregulated within the first 60 min of combined cold and osmotic stress (*i.e.* Cluster E; *see* below).

##### Protein Cluster D

Among the five clusters of the proteomic data, cluster D forms the largest group and contains 260 proteins. These proteins have a similar characteristic of abundance to those of cluster A (*i.e.* increased abundance after 4070 min of combined cold and osmotic shock onward), although their magnitude of induction was relatively weaker (Fig. 3*B*). This, in turn, means that the expression pattern of proteins in cluster D was similar to that of genes in cluster II (*see* above and Fig. 3). Taken together, the proteins in this cluster might also play an important role in promoting growth and/or survival under combined cold and osmotic stress.

Similar to protein cluster A, cluster D comprised several proteins previously known to be induced by RpoS/ECs3595 at their transcriptional level (43, 44, 49) (supplemental Table S11). This was despite the fact that these RpoS-dependent proteins are mainly associated with the response to other stresses (rather than osmotic stress in cluster A), such as acid (GadA/ECs4397, GadB/ECs2098, HdhA/ECs2327, and Slp/ECs4377) and oxidative damage (SodC/ECs2355 and SufB/ECs2390). A number of other proteins involved in adaptation to stress conditions are also present in cluster D. These include a putative catalase (ECs1652); universal stress proteins (UspA/ECs4367 and UspE/ECs1914); and YieF/ECs4650 that possesses quinone reductase activity, providing cellular protection against oxidative stress.

Cluster D also contained several proteins involved in the DNA repair system, most of whose corresponding genes are also present in cluster II (*see* above). These included a component of the RecFOR complex (RecR/ECs0525) that functions in RecA-mediated replication recovery; RecA/ECs3556, which serves as a regulatory protein to induce the SOS response to DNA damage; and subunits of the UvrABC nucleotide excision repair complex (UvrA/ECs5040 and UvrB/ECs0857). An increase in abundance of these DNA repair enzymes might indicate that DNA damage occurs upon exposure to sudden downshifts in temperature and a_w_, and that DNA damage repair is required to establish growth following the shifts. In line with this, previous studies have reported that DNA damage resulted from osmotic shock (64, 65) and cold shock (66). However, previous findings did not provide strong evidence of induced activity of the DNA repair system in either cold-adapted cells (20) or osmotically adapted cells (19).

*E. coli* upregulated a number of proteases during the period at which growth was re-established after simultaneous cold and osmotic shifts, as indicated by their presence in cluster D. These included proteins known to be responsible for the degradation of proteins (ClpA/ECs0968 and ClpP/ECs0491 and Lon/ECs0493) and peptides (Dcp/ECs2147, PrlC/ECs4370 and HslU/ECs4858), which are, respectively, important for processing irreversibly damaged or denatured proteins and peptides. This increase in the abundance of proteases may be a response for *E. coli* to cope with the increased levels of protein misfolding and aggregation caused by the downregulation of chaperones (*see* Section 'Protein cluster C') in order to re-establish growth under combined cold and osmotic stress. This agrees well with the recent study on the response of *Pseudomonas putida* during growth at low temperature (67). However, our previous study on the molecular response of *E. coli* to a temperature downshift did not reveal this trend in the expression pattern of genes and proteins in cold-adapted cells (20).

Similar to the trends observed in gene clusters I and II, cluster D contains several enzymes of the glycolysis/gluconeogenesis pathway and the TCA cycle. These include FbaA/ECs3796, PykA/ECs2564, PfkB/ECs2429, GapA/ECs2488, SucA/ECs0751, SucB/ECs0752, SucC/ECs0753, SucD/ECs0754, IcdA/ECs1608, FumC/ECs2317, and SdhA/ECs0748. The upregulation of several proteins involved in the major processes of carbohydrate metabolism and energy generation from 4070 min after applying combined cold and osmotic shifts onward, may be to compensate for the reduced activity of these processes under such stress, or may reflect a high level of energy production together with an increase in the level of precursors for the biosynthesis of various macromolecules. The latter also agrees well with the period from when these changes became apparent, suggesting that growth might have already resumed from phase II of the growth curve onward (Fig. 1). In support of this, previous studies have also demonstrated an increase in the metabolic activity of *E. coli* during exponential growth under the same stress condition (14 °C a_w_ 0.967) (18).

Cluster D contains a number of proteins involved in the biosynthesis of various amino acids. Among these proteins are those involved in the biosynthesis of isoleucine and valine (IlvA/ECs4706, IlvB/ECs4612 and IlvD/ECs4705), and histidine (HisA/ECs2825, HisB/ECs2823, HisC/ECs2822, HisD/ECs2821, HisH/ECs2824, and HisI/ECs2827). This agrees well with the trend observed in gene cluster II (*see* above). Taken together, an increase in the cellular content of these amino acids may reflect their importance in mediating growth and/or survival of *E. coli* under combined cold and osmotic stress. This is consistent with previous studies, reporting that an increased level of specific amino acids can aid tolerance to certain stress conditions (68, 69). In keeping with this, Kocharunchitt *et al.* (18) reported the same response of *E. coli* during steady-state growth at 14 °C a_w_ 0.967.

Cluster D also contained components of the min system (MinD/ECs1669 and MinE/ECs1668) that is responsible for the correct placement of the division site (the Z-ring septum) at mid-cell; MreB/ECs4123, which functions in proper chromosome segregation; essential cell division proteins (FtsI/ECs0098 and FtsI/ECs0088); and ZapA/ECs3781, which promotes the formation of the Z-ring. The abundance characteristics of these cell division-related proteins indicates that growth has been established from 4070 min of combined cold and osmotic treatment onward. This further supports the hypothesis derived from the growth study, that *E. coli* might have already resumed growth from phase II of the growth curve onward, although the physiological basis of the subsequent period, in which bacterial numbers were unchanged, remains to be elucidated (*i.e.* during phase III; *see* above).

##### Protein Cluster E

Cluster E represents 118 proteins whose abundance was transiently induced within the first 60 min after simultaneous cold and osmotic shifts (Fig. 3*B*). The transient induction of these proteins indicates that their physiological functions are part of the initial response of *E. coli* to the shifts (*i.e.* an “emergency” response). Specifically, many proteins of this cluster have previously been shown to be involved in the RpoE regulon that controls protein misfolding in the cell envelope (60, 61, 70). These included RseC/ECs3436, which is a positive regulator of RpoE activity; outer-membrane protein OmpX/ECs0892; PqiB/ECs1035 whose transcription is induced in response to superoxide generators; Imp, which has functions in the proper assembly of lipopolysaccharides at the surface of the outer membrane; YaeT/ECs0179 that is responsible for outer membrane protein biogenesis; and a protein with undefined functions (YjeP/ECs5138), despite that RpoE/ECs3439 was not detected in the present study. Similarly, the T-profiler analysis also revealed a significant induction of the RpoE regulon immediately after the shifts (Fig. 3*B*). Activation of this regulon probably serves as a multipurpose emergency response to attempt to repair protein misfolding at the cell surface. The apparent response of *E. coli* to combined cold and osmotic shifts through a transient induction of the RpoE regulon is also in keeping with previous studies on the cellular response of *E. coli* to an abrupt downshift in temperature (20, 71) and a_w_ (19, 72).

Upon simultaneous cold and osmotic shifts, *E. coli* transiently increased abundance of several proteins associated with the transport system for metallic cation, iron-siderophore and vitamin B12, as evident by their presence in cluster E. A component of the TonB-ExbBD energy transducing system (ExbB/ECs3890) that provides the energy source required for the uptake of iron-siderophore complexes and vitamin B12 across the outer membrane was observed. This was accompanied by the presence of FhuA/ECs0154, FhuC/ECs0155, FhuD/ECs0156, and FepA/ECs0623, which are respectively subunits of the TonB-dependent uptake system for iron (III) hydroxamate and ferric enterobactin. CirA/ECs3047 and Fiu/ECs0883 that act as receptors for siderophores and colicin are also grouped in this cluster. It has previously been reported that the induced activity of iron uptake systems is strongly linked with an increase in the cellular levels of hydrogen peroxide (H_2_O_2_) and superoxide (O_2_^−^). The high levels of these reactive oxygen species (ROS) can induce the oxidative stress response (68). Therefore, the apparent enhancement of iron uptake indicates that *E. coli* may suffer from oxidative damage under conditions of combined cold and osmotic stress. This likely explains the upregulation of a number of oxidative stress-responsive proteins during growth under the stress conditions (*see* Sections Cluster A and Cluster D). Furthermore, a wide range of other transport systems were found to be members of this cluster. These included those proteins involved in the uptake of lipoprotein (LolCE/ECs1494 and LolE/ECs1496), methionine (MetIN/ECs0200, and MetN/ECs0201), and nucleoside (Tsx/ECs0464).

Cluster E also contains a group of proteins involved in chemotaxis and motility (*i.e.* FliF/ECs2676, FliF/ECs2677, FlgC/ECs1452, and FlgI/ECs1458). In keeping with this, it has previously been demonstrated that osmotic shock alone (from a_w_ 0.993 to a_w_ 0.967) but not cold shock alone (from 35 °C to 14 °C) immediately induced the expression of chemotaxis and motility elements (19, 20). This has led to the hypothesis that upregulation of proteins involved in chemotaxis and motility may be because of the effects of a sudden downshift in a_w_ rather than in temperature, and that the presence of NaCl may stimulate this response. Wecker *et al.* (2009) also suggests that *E. coli* cells adopt this initial response to move away from the stress condition (73).

##### Carbon Source Utilization

**Fig. 4.**
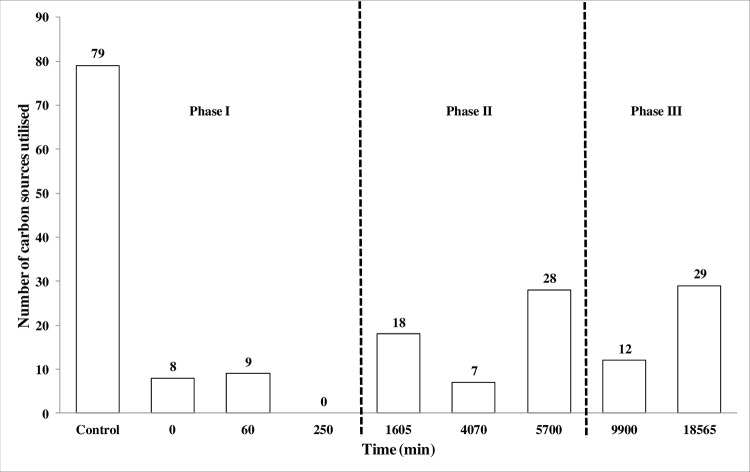
**Number of Biolog PM1 and PM2A plate carbon compounds utilized by *E. coli* O157 Sakai during a sudden downshift in temperature and water activity from 35 °C a_w_ 0.993 to 14 °C a_w_ 0.967.**

The results of the parallel Biolog study aligns with growth resuming from Phase II onwards, as we observe an increase in the number of carbon sources utilized (Fig. 4). Under the “optimal” reference conditions of 35 °C and a_w_ 0.993, *E. coli* utilized 42% (79/190) of the total number of carbon sources present on the Biolog PM1 and PM2A plates (Fig. 4). Upon a sudden downshift in temperature and water activity, the number of carbon sources utilized plummeted to 0 at 250 min (Fig. 4). Although the number of carbon sources utilized increased to 18 at 1605 min, a wavering increase was observed in the number of carbon sources utilized until a final count of 29 at 18565 min. All 29 carbon sources utilized at 18,565 min were also utilized under “optimal” conditions. The number of carbon sources utilized dropped to 7 at 4070 min, which aligns with the time at which the bacterial population underwent a second decrease and subsequent “exponential-like” phase of growth (Phase 2; Fig. 1). At 9900 min the number of carbon sources utilized dropped to 12, aligning with the time at which the population appeared to remain constant at a similar level to the starting numbers until “true exponential” phase growth was established (Phase 3; Fig. 1). However, as previously discussed, positive *T*-value results were observed for the TCA cycle and pentose phosphate pathway across these time points and there was no downregulation of genes and proteins involved in carbohydrate transport (supplemental Table S14).

In a previous report, Biolog GN2 MicroPlates were used to study the metabolism of 95 substrates by strains with distinct ratios of the general stress response sigma factor (RpoS) and the housekeeping-metabolic sigma factor (RpoD) (74). King *et al.* (74) demonstrated that strains of *E. coli* with higher RpoS levels were more resistant to external stress but metabolized fewer substrates, whereas strains with lower RpoS levels had broader nutritional capabilities. Not surprisingly, during a temperature and water activity downshift we observed a steady increase in the level of expression of *rpoS* (increases from 60 to 9900 min, slight decrease at 18565 min) and RpoS (increases from 5700 to 18565 min) (Supplemental Table S11). The Biolog study also revealed that the nutritional versatility of *E. coli* was reduced at 250 min (Fig. 4), corresponding to the time at which significant downregulation in the expression level of RpoD (log_2_ expression ratio = −5.78; supplemental Table S11) was observed. In our previous study, RpoD was also shown to be downregulated under steady-state conditions of high osmolarity (25 °C a_w_ 0.967 and 14 °C a_w_ 0.967), which was estimated to be a result of the decreased growth rate (18). Further work is required to determine the biological significance of these results and whether downregulation of RpoD under these conditions enables *E. coli* to redirect cellular energy toward repair and recovery.

##### Concluding Remarks

Under conditions of combined low temperature and water activity stresses, *E. coli* showed a complex pattern of growth behavior, including **“**loss of culturability and recovery” phases. Analysis of the associated transcriptomic and proteomic data sets pinpoint a number of factors that may be responsible for, or contribute to, the complex growth behavior of *E. coli* when exposed to such stresses. Specifically, the RpoE regulon was activated during the initial loss and subsequent recovery of culturability (during adaptation phase). This implies that RpoE plays a critical role as an emergency response for cells to repair protein misfolding in the cell envelope, possibly enabling them to survive under these stress conditions. The downregulation of molecular chaperones and those proteins associated with responses to oxidative damage during growth arrest, may be one of the contributing factors to the prolonged growth arrest of *E. coli* after the shifts. In addition, the reduction in carbon source utilization may indicate the disruption of energy-generating processes, which may further contribute to the growth arrest of *E. coli*. Finally, growth under combined cold and osmotic stress was found to activate the master stress regulator RpoS and the Rcs system-controlled colanic acid biosynthesis, as well as to increase expression of several genes and proteins with diverse functions, including those involved in the DNA repair system, the degradation of proteins and peptides, amino acid biosynthetic pathways and the major processes of carbohydrate catabolism and energy generation (*i.e.* the glycolysis/gluconeogenesis pathway and the TCA cycle). This reflects their important functions in establishing and promoting growth and/or survival under these stress conditions. The results presented in this study provide an insight into the opportunities for further studies, to ascertain which specific responses can be targeted in controlling or eliminating *E. coli* during carcass chilling or during exposure to similar processing and storage conditions.

## Supplementary Material

Supplemental Data

## Footnotes

^1^ The abbreviations used are:

GT: generation time
LT: lag time
AT: adaptation time
RLT: relative lag time
RMA: robust multiarray analysis
NSAF: normalized spectral abundance factor.

## References

[1] Pennington H. (2010) Escherichia coli O157. Lancet 376, 1428–1435 20971366 10.1016/S0140-6736(10)60963-4

[2] Rangel J. M., Sparling P. H., Crowe C., Griffin P., and Swerdlow D. L. (2005) Epidemiology of *Escherichia coli* O157:H7 outbreaks, United States, 1982–2002. Emerg. Infect. Dis. 11, 603–609 15829201 10.3201/eid1104.040739 PMC3320345

[3] Cagney C., Crowley H., Duffy G., Sheridan J. J., Brien S. O., Carney E., Anderson W., McDowell D. A., Blair I. S., and Bishop R. H. (2004) Prevalence and numbers of *Escherichia coli* O157:H7 in minced beef and beef burgers from butcher shops and supermarkets in the Republic of Ireland. Food Microbiol. 21, 203–212

[4] Elder R. O., Keen J. E., Siragusa G. R., Barkocy-Gallagher G. A., Koohmaraie M., and Laegreid W. W. (2000) Correlation of enterohemorrhagic *Escherichia coli* O157 prevalence in feces, hides, and carcasses of beef cattle during processing. Proc. Natl. Acad. Sci. U.S.A. 97, 2999–3003 10725380 10.1073/pnas.060024897 PMC16181

[5] McClure P. (2000) The impact of *E. coli* O157 on the food industry. World J. Microbiol. Biotechnol. 16, 749–755

[6] Soon J. M., Chadd S. A., and Baines R. N. (2011) Escherichia coli O157:H7 in beef cattle: on farm contamination and pre-slaughter control methods. Anim. Health Res. Rev. 12, 197–211 22152293 10.1017/S1466252311000132

[7] Hussein H. S. (2007) Prevalence and pathogenicity of Shiga toxin-producing *Escherichia coli* in beef cattle and their products. J. Anim. Sci. 85, E63–E72 17060419 10.2527/jas.2006-421

[8] Ross T., Ratkowsky D. A., Mellefont L. A., and McMeekin T. A. (2003) Modelling the effects of temperature, water activity, pH and lactic acid concentration on the growth rate of *Escherichia coli*. Int. J. Food Microbiol. 82, 33–43 12505458 10.1016/s0168-1605(02)00252-0

[9] Lovett D. A. (1978) Water transport in the surface adipose tissue of beef and mutton. Int. J. Refrig. 1, 225–228

[10] Salter M. A. (1998) Effects of temperature and water activity on *Escherichia coli* in relation to beef carcasses. University of Tasmania

[11] Gill C. O., and Bryant J. (1997) Assessment of the hygienic performances of two beef carcass cooling processes from product temperature history data or enumeration of bacteria on carcass surfaces. Food Microbiol. 14, 593–602

[12] Lenahan M., Crowley H., O'Brien S. B., Byrne C., Sweeney T., and Sheridan J. J. (2009) The potential use of chilling to control the growth of Enterobacteriaceae on porcine carcasses and the incidence of *E. coli* O157:H7 in pigs. J. Appl. Microbiol. 106, 1512–1520 19187133 10.1111/j.1365-2672.2008.04112.x

[13] Anonymous. (2007) Beef carcase chilling - opportunities for customising programs. Meat Technol. Update 2/ 07, 1–4

[14] Mellefont L., Kocharunchitt C., and Ross T. (2015) Combined effect of chilling and desiccation on survival of *Escherichia coli* suggests a transient loss of culturability. Int. J. Food Microbiol. 208, 1–10 26004386 10.1016/j.ijfoodmicro.2015.04.024

[15] Bacon R. T., Belk K. E., Sofos J. N., Clayton R. P., and Reagan J. O. S. (2000), G.C Microbial populations on animal hides and beef carcasses at different stages of slaughter in plants employing multiple-sequential interventions for decontamination. J. Food Protect. 63, 1080–1086 10.4315/0362-028x-63.8.1080 10945584

[16] Thomas J. D., Allen D. M., Hunt M. C., and Kastner C. L. (1997) Nutritional regime, post-slaughter conditioning temperature, and vacuum packing effects on bacteriology of beef carcasses and retail meat cuts. J. Food Protect. 40, 678–682 10.4315/0362-028X-40.10.678 30736231

[17] Greig J., Waddell L., Wilhelm B., Wilkins W., Bucher O., Parker S., and Rajić A. (2012) The efficacy of interventions applied during primary processing on contamination of beef carcasses with *Escherichia coli*: A systematic review-meta-analysis of the published research. Food Control 27, 385–397

[18] Kocharunchitt C., King T., Gobius K., Bowman J. P., and Ross T. (2012) Integrated Transcriptomic and Proteomic Analysis of the Physiological Response of *Escherichia coli* O157:H7 Sakai to Steady-state Conditions of Cold and Water Activity Stress. Mol. Cell. Proteomics 11, 1–16 10.1074/mcp.M111.009019 PMC3270098 22008207

[19] Kocharunchitt C., King T., Gobius K., Bowman J. P., and Ross T. (2014) Global Genome Response of *Escherichia coli* O157:H7 Sakai during Dynamic Changes in Growth Kinetics Induced by an Abrupt Downshift in Water Activity. PLoS ONE 9, e90422 24594867 10.1371/journal.pone.0090422 PMC3940904

[20] King T., Kocharunchitt C., Gobius K., Bowman J. P., and Ross T. (2014) Global Genome Response of *Escherichia coli* O157:H7 Sakai during Dynamic Changes in Growth Kinetics Induced by an Abrupt Temperature Downshift. PLoS ONE 9, e99627 24926786 10.1371/journal.pone.0099627 PMC4057180

[21] Robinson T. P., Ocio M. J., Kaloti A., and Mackey B. M. (1998) The effect of growth environment on the lag phase of *Listeria monocytogenes*. Int. J. Food Microbiol. 44, 83–92 9849786 10.1016/s0168-1605(98)00120-2

[22] Mellefont L. A., and Ross T. (2003) The effect of abrupt shifts in temperature on the lag phase duration of *Escherichia coli* and *Klebsiella oxytoca*. Int. J. Food Microbiol. 83, 295–305 12745234 10.1016/s0168-1605(02)00378-1

[23] Irizarry R. A., Hobbs B., Collin F., Beazer-Barclay Y. D., Antonellis K. J., Scherf U., and Speed T. P. (2003) Exploration, normalization, and summaries of high density oligonucleotide array probe level data. Biostatistics 4, 249–264 12925520 10.1093/biostatistics/4.2.249

[24] Benjamini Y., and Hochberg Y. (1995) Controlling the false discovery rate: A practical and powerful approach to multiple testing. J. R. Stat. Soc. 57, 289–300

[25] Rauch A., Bellew M., Eng J., Fitzgibbon M., Holzman T., Hussey P., Igra M., Maclean B., Lin C. W., Detter A., Fang R., Faca V., Gafken P., Zhang H., Whitaker J., States D., Hanash S., Paulovich A., and McIntosh M. W. (2006) Computational Proteomics Analysis System (CPAS): An Extensible, Open-Source Analytic System for Evaluating and Publishing Proteomic Data and High Throughput Biological Experiments. J. Proteome Res. 5, 112–121 16396501 10.1021/pr0503533

[26] Craig R., and Beavis R. C. (2004) TANDEM: matching proteins with tandem mass spectra. Bioinformatics 20, 1466–1467 14976030 10.1093/bioinformatics/bth092

[27] Keller A., Nesvizhskii A. I., Kolker E., and Aebersold R. (2002) Empirical statistical model to estimate the accuracy of peptide identifications made by MS/MS and database search. Anal. Chem. 74, 5383–5392 12403597 10.1021/ac025747h

[28] Nesvizhskii A. I., Keller A., Kolker E., and Aebersold R. (2003) A statistical model for identifying proteins by tandem mass spectrometry. Anal. Chem. 75, 4646–4658 14632076 10.1021/ac0341261

[29] Choi H., and Nesvizhskii A. I. (2008) False Discovery Rates and Related Statistical Concepts in Mass Spectrometry-Based Proteomics. J. Proteome Res. 7, 47–50 18067251 10.1021/pr700747q

[30] Liu H., Sadygov R. G., and Yates JR III (2004) A model for random sampling and estimation of relative protein abundance in shotgun proteomics. Anal. Chem. 76, 4193–4201 15253663 10.1021/ac0498563

[31] Zybailov B. L., Mosley A. L., Sardiu M. E., Coleman M. K., Florens L., and Washburn M. P. (2006) Statistical analysis of membrane proteome expression changes in *Saccharomyces cerevisiae*. J. Proteome Res. 5, 2339–2347 16944946 10.1021/pr060161n

[32] Pham T. V., Piersma S. R., Warmoes M., and Jimenez C. R. (2010) On the beta-binomial model for analysis of spectral count data in label-free tandem mass spectrometry-based proteomics. Bioinformatics 26, 363–369 20007255 10.1093/bioinformatics/btp677

[33] Boorsma A., Foat B. C., Vis D., Klis F., and Bussemaker H. J. (2005) T-profiler: scoring the activity of predefined groups of genes using gene expression data. Nucleic Acids Res. 33, w592–w595 15980543 10.1093/nar/gki484 PMC1160244

[34] Zhou K., Zhou L., Lim Q. E., Zou R., Stephanopoulos G., Too H-P. (2011) Novel reference genes for quantifying transcriptional responses of *Escherichia coli* to protein overexpression by quantitative PCR. BMC Mol. Biol. 12 10.1186/1471-2199-12-18 PMC3110127 21513543

[35] Rozen S., and Skaletsky H. (2000) Primer3 on the WWW for general users and for biologist programmers. Methods Mol. Biol. 132, 365–386 10547847 10.1385/1-59259-192-2:365

[36] Pfaffl M. W. (2001) A new mathematical model for relative quantification in real-time RT-PCR. Nucleic Acids Res. 29, e45 11328886 10.1093/nar/29.9.e45 PMC55695

[37] Vizcaíno J. A., Deutsch E. W., Wang R., Csordas A., Reisinger F., Rios D., Dianes J. A., Sun Z., Farrah T., and Bandeira N. (2014) ProteomeXchange provides globally coordinated proteomics data submission and dissemination. Nat. Biotechnol. 32, 223–226 24727771 10.1038/nbt.2839 PMC3986813

[38] Baker P. R., and Chalkley R. J. (2014) MS-viewer: a web-based spectral viewer for proteomics results. Mol. Cell. Proteomics 13, 1392–1396 24591702 10.1074/mcp.O113.037200 PMC4014294

[39] Mellefont L. A., McMeekin T. A., and Ross T. (2003) The effect of abrupt osmotic shifts on the lag phase duration of foodborne bacteria. Int. J. Food Microbiol. 83, 281–293 12745233 10.1016/s0168-1605(02)00377-x

[40] Gygi S. P., Rochon Y., Franza B. R., and Aebersold R. (1999) Correlation between Protein and mRNA Abundance in Yeast. Mol. Cell. Biol. 19, 1720–1730 10022859 10.1128/mcb.19.3.1720 PMC83965

[41] Rossouw D., van den Dool A. H., Jacobson D., and Bauer F. F. (2010) Comparative Transcriptomic and Proteomic Profiling of Industrial Wine Yeast Strains. Appl. Environmental Microbiol. 76, 3911–3923 10.1128/AEM.00586-10 PMC2893464 20418425

[42] Raychaudhuri S., Stuart J. M., and Altman R. B. (2000) principal components analysis to summarize microarray experiments: application to sporulation time series. Pacific Symposium on Biocomputing, 455–466 10.1142/9789814447331_0043 PMC2669932 10902193

[43] Weber H., Polen T., Heuveling J., Wendisch V. F., and Hengge R. (2005) Genome-wide analysis of the general stress response network in *Escherichia coli*: σ^S^-dependent genes, promoters, and sigma factor selectivity. J. Bacteriol. 187, 1591–1603 15716429 10.1128/JB.187.5.1591-1603.2005 PMC1063999

[44] Patten C. L., Kirchhof M. G., Schertzberg M. R., Morton R. A., and Schellhorn H. E. (2004) Microarray analysis of RpoS-mediated gene expression in *Escherichia coli* K-12. Mol. Genet. Genomics 272, 580–591 15558318 10.1007/s00438-004-1089-2

[45] Francez-Charlot A., Laugel B., Van Gemert A., Dubarry N., Wiorowski F., Castanié-Cornet M-P, Gutierrez C., and Cam K. (2003) RcsCDB His-Asp phosphorelay system negatively regulates the flhDC operon in *Escherichia coli*. Mol. Microbiol. 49, 823–832 12864862 10.1046/j.1365-2958.2003.03601.x

[46] Thieringer H. A., Jones P. G., and Inouye M. (1998) Cold shock and adaptation. Bioessays 20, 49–57 9504047 10.1002/(SICI)1521-1878(199801)20:1<49::AID-BIES8>3.0.CO;2-N

[47] Yamanaka K. (1999) Cold shock response in *Escherichia coli*. J. Mol. Microbiol. Biotechnol. 1, 193–202 10943550

[48] Etchegaray J-P, and Inouye M. (1999) CspA, CspB, and CspG, major cold shock proteins of *Escherichia coli*, are induced at low temperature under conditions that completely block protein synthesis. J. Bacteriol. 181, 1827–1830 10074075 10.1128/jb.181.6.1827-1830.1999 PMC93581

[49] Lacour S., and Landini P. (2004) σ^S^-dependent gene expression at the onset of stationary phase in *Escherichia coli*: function of σ^S^-dependent genes and identification of their promoter sequences. J. Bacteriol. 186, 7186–7195 15489429 10.1128/JB.186.21.7186-7195.2004 PMC523212

[50] Weber A., and Jung K. (2002) Profiling Early Osmostress-Dependent Gene Expression in *Escherichia coli* Using DNA Macroarrays. J. Bacteriol. 184, 5502–5507 12218039 10.1128/JB.184.19.5502-5507.2002 PMC135335

[51] Weber A., Kögl S. A., and Jung K. (2006) Time-Dependent Proteome Alterations under Osmotic Stress during Aerobic and Anaerobic Growth in *Escherichia coli*. J. Bacteriol. 188, 7165–7175 17015655 10.1128/JB.00508-06 PMC1636219

[52] Moen B., Oust Janbu A., Langsrud S., Langsrud Ø Hobman J., L, Constantinidou C., Kohler A., and Rudi K. (2009) Global responses of *Escherichia coli* to adverse conditions determined by microarrays and FT-IR spectroscopy. Can. J. Microbiol. 55, 714–728 19767843 10.1139/w09-016

[53] Hagiwara D., Sugiura M., Oshima T., Mori H., Aiba H., Yamashino T., and Mizuno T. (2003) Genome-wide analyses revealing a signaling network of the RcsC-YojN-RcsB phosphorelay system in *Escherichia coli*. J. Bacteriol. 185, 5735–5746 13129944 10.1128/JB.185.19.5735-5746.2003 PMC193970

[54] Ophir T., and Gutnick D. L. (1994) A role for exopolysaccharides in the protection of microorganisms from desiccation. Appl. Environmental Microbiol. 60, 740–745 10.1128/aem.60.2.740-745.1994 PMC201377 16349202

[55] Chen J., Lee S. M., and Mao Y. (2004) Protective effect of exopolysaccharide colanic acid of *Escherichia coli* O157:H7 to osmotic and oxidative stress. Int. J. Food Microbiol. 93, 281–286 15163584 10.1016/j.ijfoodmicro.2003.12.004

[56] Mao Y., Doyle M. P., and Chen J. (2006) Role of colanic acid exopolysaccharide in the survival of enterohaemorrhagic *Escherichia coli* O157:H7 in simulated gastrointestinal fluids. Lett. Appl. Microbiol. 42, 642–647 16706906 10.1111/j.1472-765X.2006.01875.x

[57] Danese P. N., Pratt L. A., and Kolter R. (2000) Exopolysaccharide production is required for development of *Escherichia coli* K-12 biofilm architecture. J. Bacteriol. 182, 3593–3596 10852895 10.1128/jb.182.12.3593-3596.2000 PMC101973

[58] Sledjeski D. D., and Gottesman S. (1996) Osmotic shock induction of capsule synthesis in *Escherichia coli* K-12. J. Bacteriol. 178, 1204–1206 8576059 10.1128/jb.178.4.1204-1206.1996 PMC177786

[59] Mackey B. M., and Derrick C. M. (1986) Peroxide sensitivity of cold-shocked *Salmonella typhimurium* and *Escherichia coli* and its relationship to minimal medium recovery. J. Appl. Bacteriol. 60, 501–511 3017912 10.1111/j.1365-2672.1986.tb01089.x

[60] Alba B. M., and Gross C. A. (2004) Regulation of the *Escherichia coli* σ^E^-dependent envelope stress response. Mol. Microbiol. 52, 613–619 15101969 10.1111/j.1365-2958.2003.03982.x

[61] Nonaka G., Blankschien M., Herman C., Gross C. A., and Rhodius V. A. (2006) Regulon and promoter analysis of the *E. coli* heat-shock factor, σ^32^, reveals a multifaceted cellular response to heat stress. Genes Dev. 20, 1776–1789 16818608 10.1101/gad.1428206 PMC1522074

[62] Strocchi M., Ferrer M., Timmis K. N., and Golyshin P. N. (2006) Low temperature-induced systems failure in *Escherichia coli*: insights from rescue by cold-adapted chaperones. Proteomics 6, 193–206 16302275 10.1002/pmic.200500031

[63] Kim Y-H, Han K. Y., Lee K., and Lee J. (2005) Proteome response of *Escherichia coli* fed-batch culture to temperature downshift. Appl. Microbiol. Biotechnol. 68, 786–793 15983803 10.1007/s00253-005-0053-3

[64] Fitt P. S., Sharma N., and Ball D. (1992) Induction of error-free DNA repair in *Escherichia coli* by Nonmutagenic Stress. Curr. Microbiol. 24, 251–256

[65] Ribeiro G. F., Côrte-Real M., and Johansson B. (2006) Characterization of DNA damage in yeast apoptosis induced by hydrogen peroxide, acetic acid, and hyperosmotic shock. Mol. Biol. Cell 17, 4584–4591 16899507 10.1091/mbc.E06-05-0475 PMC1635349

[66] Ray M. K. (2006) Cold-stress response of low temperature adapted bacteria. In: Sreedhar A. S., and Srinivas U. K., eds. Stress response: a molecular biology approach, pp. 1–23, Research Signpost, Trivandrum, India

[67] Fonseca P., Moreno R., and Rojo F. (2011) Growth of *Pseudomonas putida* at low temperature: global transcriptomic and proteomic analyses. Environmental Microbiol. Reports 3, 329–339 10.1111/j.1758-2229.2010.00229.x 23761279

[68] Horinouchi T., Tamaoka K., Furusawa C., Ono N., Suzuki S., Hirasawa T., Yomo T., and Shimizu H. (2010) Transcriptome analysis of parallel-evolved *Escherichia coli* strains under ethanol stress. BMC Genomics 11, 579 20955615 10.1186/1471-2164-11-579 PMC3091726

[69] Jozefczuk S., Klie S., Catchpole G., Szymanski J., Cuadros-Inostroza A., Steinhauser D., Selbig J., and Willmitzer L. (2010) Metabolomic and transcriptomic stress response of *Escherichia coli*. Mol. Syst. Biol. 6, 364 20461071 10.1038/msb.2010.18 PMC2890322

[70] Rhodius V. A., Suh W. C., Nonaka G., West J., and Gross C. A. (2006) Conserved and Variable Functions of the σ^E^ Stress Response in Related Genomes. PLos Biol. 4, 43–59 10.1371/journal.pbio.0040002 PMC1312014 16336047

[71] Polissi A., De Laurentis W., Zangrossi S., Briani F., Longhi V., Pesole G., and Dehò G. (2003) Changes in *Escherichia coli* transcriptome during acclimatization at low temperature. Res. Microbiol. 154, 573–580 14527658 10.1016/S0923-2508(03)00167-0

[72] Bianchi A. A., and Baneyx F. (1999) Hyperosmotic shock induces the σ^32^ and σ^E^ stress regulons of *Escherichia coli*. Mol. Microbiol. 34, 1029–1038 10594827 10.1046/j.1365-2958.1999.01664.x

[73] Wecker P., Klockow C., Ellrott A., Quast C., Langhammer P., Harder J., and Glöckner F. O. (2009) Transcriptional response of the model planctomycete *Rhodopirellula baltica* SH1^T^ to changing environmental conditions. BMC Genomics 10, 1–16 19725962 10.1186/1471-2164-10-410 PMC2754498

[74] King T., Ishihama A., Kori A., and Ferenci T. (2004) A Regulatory Trade-Off as a Source of Strain Variation in the Species *Escherichia coli*. J. Bacteriol. 186, 5614–5620 15317765 10.1128/JB.186.17.5614-5620.2004 PMC516835

