# Supplementary material for: Physiological Response of Escherichia coli O157:H7 Sakai to Dynamic Changes in Temperature and Water Activity as Experienced during Carcass Chilling
Source: Mol Cell Proteomics. 2016 Sep 11;15(11):3331–47. doi: 10.1074/mcp.M116.063065 (PMC5098033; doi:10.1074/mcp.M116.063065)
Supplement: Supplemental Data [file supp_15_11_3331__index.html]

Physiological response of Escherichia coli O157:H7 Sakai to dynamic changes in temperature and water activity as experienced during carcass chilling — Physiological Response of Escherichia coli O157:H7 Sakai to Dynamic Changes in Temperature and Water Activity as Experienced during Carcass Chilling — Response of E. coli to Temperature and Water Activity Downshift — Supplemental Data 

# Physiological Response of *Escherichia coli* O157:H7 Sakai to Dynamic Changes in Temperature and Water Activity as Experienced during Carcass Chilling

## Supplemental Data

- Supplementary Table 1 (.xlsx, 969 KB) - Supplementary Table 1
- Supplementary Table 2 (.xlsx, 577 KB) - Supplementary Table 2
